# Rapid and Efficient Conversion of Integration-Free Human Induced Pluripotent Stem Cells to GMP-Grade Culture Conditions

**DOI:** 10.1371/journal.pone.0094231

**Published:** 2014-04-09

**Authors:** Jens Durruthy-Durruthy, Sharon F. Briggs, Jason Awe, Cyril Y. Ramathal, Saravanan Karumbayaram, Patrick C. Lee, Julia D. Heidmann, Amander Clark, Ioannis Karakikes, Kyle M. Loh, Joseph C. Wu, Andrew R. Hoffman, James Byrne, Renee A. Reijo Pera, Vittorio Sebastiano

**Affiliations:** 1 Institute for Stem Cell Biology and Regenerative Medicine, Department of Obstetrics and Gynecology and Department of Genetics, Stanford University, Stanford, California, United States of America; 2 Eli and Edythe Broad Center for Regenerative Medicine & Stem Cell Research, University of California Los Angeles, Los Angeles, California, United States of America; 3 Department of Molecular and Medical Pharmacology, University of California Los Angeles, Los Angeles, California, United States of America; 4 Department of Microbiology, Immunology and Molecular Genetics, University of California Los Angeles, Los Angeles, California, United States of America; 5 Veterans Affairs Palo Alto Health Care System, Stanford University, Palo Alto, California, United States of America; 6 Department of Molecular, Cellular, and Developmental Biology, Molecular Biology Institute, University of California Los Angeles, Los Angeles, California, United States of America; 7 Stanford Cardiovascular Institute, Stanford University, Stanford, California, United States of America; 8 Department of Cardiology, Department of Medicine, Stanford University, Stanford, California, United States of America; 9 Department of Developmental Biology, Stanford University, Stanford, California, United States of America; Johns Hopkins School of Medicine, United States of America

## Abstract

Data suggest that clinical applications of human induced pluripotent stem cells (hiPSCs) will be realized. Nonetheless, clinical applications will require hiPSCs that are free of exogenous DNA and that can be manufactured through Good Manufacturing Practice (GMP). Optimally, derivation of hiPSCs should be rapid and efficient in order to minimize manipulations, reduce potential for accumulation of mutations and minimize financial costs. Previous studies reported the use of modified synthetic mRNAs to reprogram fibroblasts to a pluripotent state. Here, we provide an optimized, fully chemically defined and feeder-free protocol for the derivation of hiPSCs using synthetic mRNAs. The protocol results in derivation of fully reprogrammed hiPSC lines from adult dermal fibroblasts in less than two weeks. The hiPSC lines were successfully tested for their identity, purity, stability and safety at a GMP facility and cryopreserved. To our knowledge, as a proof of principle, these are the first integration-free iPSCs lines that were reproducibly generated through synthetic mRNA reprogramming that could be putatively used for clinical purposes.

## Introduction

Derivation of human induced pluripotent stem cells (hiPSCs) from virtually any adult tissue provides numerous opportunities for development of therapeutic strategies for multiple pathologies that involve tissue degeneration [1], [2]. Nonetheless the promise of hiPSCs in regenerative medicine relies on overcoming several hurdles. In particular, clinical application of hiPSCs derivatives necessitates a derivation protocol with minimal risk of integration of exogenous DNA, as random integration can lead to insertional mutagenesis with unpredictable effects on the quality of the cells and the potential safety after transplantation [3]. Although non-integrative DNA-based methods of inducing pluripotency including episomal vectors [4] and minicircles [5] have been developed, it is difficult to exclude the possibility of integration of very small fragments of DNA. Deep whole genome sequencing of iPSCs derived with these methods could be used to exclude genomic integration, but this is costly. Moreover, extensive passaging is required to dilute out exogenous vectors, which increases opportunities for the acquisition of mutations. Other proposed non-integrative methods like protein-based reprogramming [6] or Sendai virus [7] are very inefficient (protein-based) or require extensive passaging to remove residual viral expression (Sendai virus). Recently it has been shown that modified mRNAs can be used to efficiently derive footprint-free iPSCs [8], [9].

It is also critical that derivation of high quality hiPSCs for clinical applications should be rapid, efficient and cost effective. This is especially important considering that, after reprogramming, clonal lines require characterization and may require genome correction (in the case of genetic diseases) and differentiation to a transplantable cell type. All of these steps require extensive maintenance in culture and are therefore associated with mutagenesis that can affect quality of the cells and ability of the cells to engraft and function properly [10], [11]. It is important that methods are compatible with future uses in pathologies associated with tissue-degeneration where a fast and efficient cell therapy is crucial and cost-effective.

Finally, even after the rapid and integration-free derivation of hiPSCs, clinically compliant cell products are required for potential cell based therapies. Previous regulatory oversight suggests that two methods may be acceptable for this purpose: 1) Derivation of cells and cell products under GMP requirements and 2) Conversion of cells or cell products derived under research-grade conditions to GMP quality standards (http://www.fda.gov/downloads/drugs/guidancecomplianceregulatoryinformation/guidances/ucm070273.pdf). The latter has recently been demonstrated with a lentiviral-derived hiPSC line [12], which required that the cells be successfully transferred to a GMP facility and cultured, frozen, thawed, and expanded extensively in such an environment. Also, rigorous tests should be passed to exclude the presence of adventitious agents that could have been present in the research-grade reagents used during the cell derivation process. Thus, it is important to develop a standardized platform of iPSCs derivation that is integration-free, fast, efficient, scalable and easily transferred to GMP compliant conditions. Here, we address some of the issues that are currently associated with the use of hiPSCs in clinical research. We propose that this study provides a valuable resource for the scientific community towards developing a standardized procedure to derive hiPSCs that are putatively GMP compliant.

## Materials and Methods

### Tissue acquisition

Written approval for all somatic derivations and subsequent iPSC generation performed in this study was obtained from the Stanford University Institutional Review Board (IRB protocol 10368) and the Stanford University Stem Cell Research Oversight Committee (SCRO protocol 40), and written informed consent was obtained from each individual participant.

### Fibroblasts

BJ human fibroblast cells (passage 6) were established from normal human fetal foreskin and purchased from Stemgent (Cambridge, MA). GM13325 fibroblast cells were obtained from a 9-day old patient with DiGeorge Syndrome (purchased from Coriell, Camden, NJ). Human adult fibroblasts (HUF1 and HUF58) were derived from a healthy male (age 28) and a patient (60 years), respectively through a skin punch biopsy as previously described [13].

Human fibroblast cell lines were cultured on 0.2% gelatin (Sigma) coated wells in DMEM-FBS a culture medium consisting of Dulbecco's modified Minimal Essential Medium + GlutaMAX (DMEM), 10% fetal bovine serum (FBS), 100 U/ml penicillin and 100 μg/ml streptomycin (all Invitrogen). When 80–90% confluent, cells were passaged using TrypLE Express (Invitrogen) and re-plated at a 1∶3 dilution. Human ES cells (H9) and iPSCs were cultured in W8 medium, a culture medium consisting of DMEM/F12 supplemented with 20% Knockout Serum Replacer, 2 mM L-glutamine, 0.1 mM Non-Essential Amino Acids (NEAA), 0.1 mM 2-Mercaptoethanol (Millipore) and 10 ng /ml b-FGF. Fibroblasts were frozen in 90% FBS (Invitrogen) and 10% dimethyl sulfoxide (DMSO, Sigma). hESC and iPSC cells were frozen in Bambanker (Wako Chemicals).

### hESCs and iPSCs

For feeder-independent maintenance of human ESCs and iPSCs, basal mTeSR1 medium (STEMCELL Technologies) supplemented with 5X mTeSR1 supplement (STEMCELL Technologies) was used. Culture plates were pre-coated with growth factor reduced matrigel (BD Biosciences). Cells were passaged mechanically or enzymatically using 200 units/ml of collagenase IV or dispase (STEMCELL Technologies), washed and replated at a dilution of 1∶2 to 1∶5. Differentiated cells were removed and/or cleaned under a laminar flow dissection hood. Cultures were maintained at 37°C and 5% CO_2_ (for reprogramming experiments hypoxic conditions were applied) and medium changed every other day for fibroblast lines and every day for hESC and iPSC lines. For GMP conversion, cells were first gradually adapted to a 1∶1 blend of mTeSR1 medium (STEMCELL Technologies) supplemented with 5X mTeSR1 supplement (STEMCELL Technologies) and Nutristem (Stemgent) before they were fully converted towards a 1∶1 blend of TeSR2 (STEMCELL Technologies) and Nutristem (Stemgent). Cultures initially derived on Synthemax (Corning) or CELLstart (Invitrogen) were directly converted towards a 1∶1 blend of TeSR2 (STEMCELL Technologies) and Nutristem (Stemgent).

### Immunocytochemistry

For immunostaining cells were fixed in 4% paraformaldehyde for 10 min and washed. Cells were permeabilized with 0.2% Triton X (Sigma) in PBS for 15 min and washed 2x with PBS followed by blocking with 4% serum in PBS and 0.2% Triton X for 30 min at room temperature. Primary antibodies (diluted in blocking buffer) were incubated overnight at 4°C as follows: OCT3/4 (1∶200) -Santa Cruz Biotechnology-; SOX2 (1∶200), SSEA4 (1∶200), TRA-1-60 (1∶200) and TRA-1-81 (1∶200) –Millipore-; NANOG (1∶400) -Abcam-; alpha-fetoprotein (1∶200), TUJ1 (1∶1000) and DESMIN (1∶500) -R&D Systems-. Cells were washed 2x with PBS (5 min each) before secondary antibodies (Alexa, Invitrogen) were applied at 1∶1000 (diluted in blocking buffer) for 30 min at room temperature. Finally, cells were washed 3x with PBS (5 min each) and counter stained with DAPI (300 nM in PBS) followed by fluorescence microscopy (Olympus).

#### Immunostaining of live cells

StainAlive DyLight 488 anti-Human TRA-1-60/TRA-1-81 antibody (Stemgent) was diluted in fresh cell culture medium to a final concentration of 5 μg/ml. Old medium was aspirated and replaced with medium containing diluted antibodies. Cells were incubated for 30 min at 37°C and 5% CO_2_. The primary antibody was aspirated and cells were washed gently 2x with cell culture medium. Fresh cell culture medium was added and cells were examined under a fluorescent microscope using the appropriate filters. Cells were kept in culture after examination.

### *In vitro* transcription

Synthesis for mRNA was carried out with the MEGAscript T7 kit (Ambion) according to the manufacturer's instructions with slight modifications. A custom ribonucleoside blend was comprised of 6 mM 3′-0-Me-m7G(5′)ppp(5′)G ARCA cap analog (New England Biolabs), 7.5 mM of adenosine triphosphate and 1.5 mM of guanosine triphosphate (Ambion), 7.5 mM of 5-methylcytidine triphosphate and pseudouridine triphosphate (TriLink Biotechnologies). Reactions were incubated for 4 h at 37°C, followed by DNase treatment for 15 min at 37°C. DNase treated RNA was purified using the MEGAclear kit (Ambion). Correct RNA synthesis and RNA purification was verified and quantified using a Nanodrop (A230/A260 between 1.7–2.0) and concentration was adjusted to 100 ng/ml. RNA reprogramming cocktails were prepared by pooling individual 100 ng/μl RNA stocks to produce a 100 ng/μl total blend. Stocks were stored at −80°C. RNAse mediated RNA degradation was prevented by cleaning the working space and the instruments with RNaseZap (Ambion).

### Denaturing formaldehyde-agarose gel electrophoresis

mRNAs were analyzed to verify specificity of IVT reaction and correct size of the transcripts. A 1.5% denaturing formaldehyde-agarose gel was prepared dissolving 0.75 g agarose in 36 ml DI water, 5 ml 10X MOPS running buffer (Ambion) and 9 ml 37% formaldehyde (12.3 M, Sigma-Aldrich). mRNA samples (1 μg) were mixed with 3x the volume of Formaldehyde Loading Dye (Ambion) and 0.25 μl ethidium bromide (10 mg/ml, Bio-Rad) followed by heat denaturation for 15 min at 70°C. RNA ladder (RNA Millenium marker, Ambion) was treated like mRNA samples and used for size comparison.

### mRNA transfection

mRNA transfection was carried out with RNAiMAX (Invitrogen) according to the manufacturer's instruction in a 6-well format. Briefly, mRNA and reagent were first diluted in Opti-MEM basal medium (Invitrogen). 1.2 ug (100 ng/μl) RNA was diluted in 48 ul Opti-MEM and 6 ul RNAiMAX was diluted in 54 ul Opti-MEM. Both dilutions were combined to a total of 120 ul, briefly vortexed and incubated for 15 min at room temperature. After complex formation, mix was drop-wise dispensed to culture medium. RNA transfection was performed in Pluriton media (Stemgent) supplemented with Pluriton supplement (Stemgent) and B18R interferon inhibitor (eBioscience) at 200 ng/ml.

### Derivation of mRNA induced pluripotent stem cells (RiPSCs)

Target fibroblasts were seeded at 1×10^4^–5×10^4^ cells per well of a 6-well plate on 0.2% gelatin coated wells and cultured in Pluriton media. After 24 hours, Pluriton media was changed to NuFF (Globalstem) conditioned Pluriton media (Stemgent) supplemented with Pluriton supplement (Stemgent) and B18R (200 ng/ml, eBioscience). Cells were transferred to a low oxygen environment (5%) for higher reprogramming efficiencies before the first transfection. After 2 h of equilibration in low oxygen conditions the mRNA cocktail containing OSKMLg (*OCT3/4, SOX2, KLF4, cMYC, LIN28A, eGFP*) was transfected and repeated every 24 h until colony formation was observed. Incubation of mRNA and transfection mix with cells was carried out for 4 h. Media was replaced with fresh Pluriton media (Stemgent) containing Pluriton supplement and B18R recombinant protein (200 ng/ml) to inhibit cellular immune response.

Primary colonies were picked onto new culture dishes freshly coated with Matrigel (BD Bioscience) and media was replaced with mTeSR1 (STEMCELL Technologies) supplemented with 5X mTeSR1 supplement (STEMCELL Technologies) and Nutristem (Stemgent). Established iPSCs lines were cultured under 20% oxygen conditions. For RiPSC derivation under fully defined GMP conditions, matrigel was replaced with CELLstart and xeno-free Pluriton media (not conditioned on NuFFs). RiPSCs were derived on average within 2 weeks from the first mRNA transfection. In the case of mRNA reprogramming under GMP-conditions, cells were fully GMP-grade within 2 weeks.

### Conversion, culture and characterization under GMP conditions

Cells were gradually converted from Pluriton to mTeSR1/Nutristem as follows: 1∶0, 0.8∶0.2, 0.5∶0.5, 0.2∶0.8, 0∶1 with x:x being the ratio of Pluriton:mTeSR1/Nutristem. The ratio was altered with each daily media change. The same conversion strategy was applied for mTeSR1/Nutristem to TeSR2/Nutristem conversion. Cells were manually passaged with glass tools during the entire conversion on Synthemax (Corning), an extra cellular matrix with cell adhesion promoting peptides. Synthemax is a synthetic, non-biological, xeno-free, gamma sterilized (SAL 10^−6^) substrate and quality tested. Pluriton, Nutristem and TeSR2 are chemically defined and xeno-free cell culture media, while mTeSR1 contains animal proteins. All media can be used for feeder-free culturing of hESCs/iPSCs.

Cells that were converted to xeno-free conditions were then transferred to the UCLA GMP compatible facility, expanded under xeno-free conditions and cryobanked for future applications. Fully converted cells (2–4 weeks from start of conversion) were subject to characterization including immunocytochemistry, sterility tests (Gram positive/negative bacteria, fungi, mycoplasma), karyotyping and STR analysis (Cell Line Genetics). The UCLA GMP-compliant facility is built with ISO 7/Class 10,000 clean room and ISO 5/Class 100 biosafety cabinet (BSC). The equipment in the facility is monitored on a daily basis and SOPs are followed for cleaning and operation. External certified vendors calibrate the biosafety cabinets and monitor air as per ISO standards and perform viable particle count in clean rooms and BSCs. Temperature and gases are also continuously monitored in the equipment through a wireless network system operated by contract vendors. Periodic calibrations are done to ensure functioning of the equipment as stipulated. External vendors also perform calibration and qualification of pipettes. Testing of adventitious agents is done by clinically certified laboratories. Our in-house qualification method involves cleaning and decontamination for water baths, incubators, refrigerators, freezers, and centrifuges.

### Gene expression analysis

Gene expression analysis was performed using the Fluidigm system (San Francisco, CA) according to the manufacture's protocol “Single-Cell Gene Expression Using EvaGreen DNA Binding Dye” with some modifications for bulk samples. Briefly, purchased forward and reverse primer pairs were mixed together to a final concentration of 20 μM. All primer pairs were pooled together at a final concentration of 200 nM each for pre-amplification. CellsDirect 2x Reaction Mix (Invitrogen), SuperScript III RT Platinum Taq Mix (Invitrogen), 4X Primer Mix (200 nM) and TE buffer were prepared at total volume of 9 μl. Cells in bulk (10–200 cells) were manually picked under the dissection hood added in 1 μl to each reaction and the following thermal cycling protocol was set: Reverse Transcription −50°C for 15 min, Inactivate RT/Activate Taq - 95°C for 2 min, 18 Cycles - 95°C for 15 sec and 60°C for 4 min, 4°C for infinite. ExoSAP-IT treatment removed unused material and was performed at 37°C for 15 min (Digest) and 80°C for 15 min (Inactivation). Reaction was diluted 1∶5 in TE buffer and stored at −20°C or immediately used for Sample Pre-Mix. Sample Pre-Mix, Samples and Assay Mix were prepared according to the protocol. The Fluidigm chip was primed and loaded with the assay and sample mix. Data were collected and analyzed using the Fluidigm Data Collection Software v.3.0.2. Unsupervised hierarchical clustering analysis was performed with R.

### EB formation and spontaneous in vitro differentiation

Using AggreWell 400 (STEMCELL Technologies) cells were harvested and prepared according to the manufacturer's instructions and incubated for 2 days in the AggreWell plates. Cells were then seeded into 2 gelatin-coated wells of a 24 well plate containing DMEM +20% FBS. Medium was changed every 2–3 days for up to 2 weeks. Cells were fixed in 4% paraformaldehyde for staining of markers representative for the three germ layers.

### Directed *in vitro* differentiation

Definitive endoderm: hiPSCs were passaged as very fine clumps using enzymatic dissociation (Accutase, Life Technologies) and the following day, were differentiated towards definitive endoderm for 48 hours using defined, serum-free monolayer conditions. In brief, hiPSCs were washed, differentiated towards anterior primitive streak for 24 hours, washed once more and then sequentially differentiated towards definitive endoderm. To ascertain production of definitive endoderm, differentiated populations were stained for cell-surface markers CXCR4 and PDGFRA and subject to FACS analysis [14].

Neuroectoderm: hiPSC medium was switched to differentiation medium (DMEM-N2-B27 containing 10 μm SB431542 and 2 μm Dorsomorphin). Media was changed daily for a total of 7–10 days.

Cardiomyocytes: Cells were differentiated as previously reported [15]. Briefly, hiPSC were dissociated and cultured in suspension. Cardiomyocyte differentiation was induced by sequential exposure to growth factors (BMP4 and Activin-A) and small molecules (IWR-1) in a chemical defined media (StemPro34, Life technologies). Cardiomyocytes were immunostained with an anti-Cardiac Troponin T antibody (1∶200, Thermofisher Scientific) for immunofluorescence analysis.

### Teratoma formation

For each graft, one iPSCs line from one confluent 10 cm dish was enzymatically harvested, washed and resuspended in a 1.5 ml tube containing 300 ul 30% Matrigel diluted in PBS. Cells were then injected subcutaneously into female SCID mice (Charles River Laboratories International, Inc.). Visible tumors were dissected 3–8 weeks post-transplantation and fixed overnight with 4% PFA diluted in PBS. Fixed samples were sent to AML Laboratories (Baltimore, MD) for paraffin embedding, sectioning and staining with hematoxylin and eosin. Sections were then examined for the presence of tissue representatives of all three germ layers.

### Statistical Analysis

Analysis of variance (ANOVA) statistical comparisons were performed using GraphPad Prism (La Jolla, CA) and SPSS (IBM, Armonk, NY) with statistical significance set at p<0.05. Student's two-tailed t test was used to determine statistical significance for data generated from Fluidigm gene expression assays.

## Results

### Derivation of RiPSC with an optimized protocol

**Figure 1:**
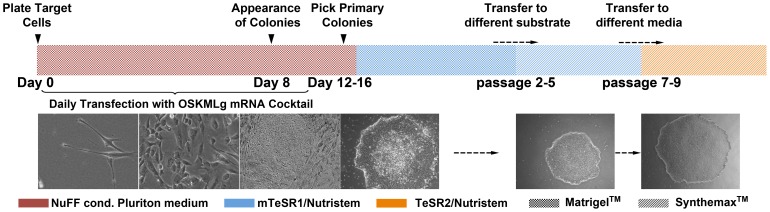
Overview of optimized derivation of mRNA-induced pluripotent stem cells and conversion to GMP clinical-grade conditions. Overview of feeder-free reprogramming with modified mRNA. Morphology tracking of reprogrammed human fibroblasts during the course of 10–16 days. Fibroblasts show early epitheliod morphology and small cluster formation that lead into small hES cell like colonies. Small colonies grow in size and become mature RiPSC colonies. Derived lines were converted to GMP compatible matrix and culture conditions. See also Figure S1 and Table S1.

Four genetically-distinct lines, from both newborn foreskin and adult dermal fibroblasts, were derived. Two lines (named HUF1 and HUF58) were established in the lab from a punch skin biopsy of a 28 year old healthy adult male (46 XY) and a 60 year old adult male (46 XY, chromosome 2 pericentric inversion), respectively. The third line (named GM13325) was purchased from Coriell and originated from a 9 day old patient with DiGeorge Syndrome (46 XX) and the fourth, BJ fibroblasts (46 XY), was purchased from Stemgent and was derived from a newborn (Table S1). For reprogramming, we used an optimized and simplified version of previously published protocols of RiPSC derivation [8], [9]. We observed that one of the most crucial parameters to determine the success of reprogramming (even in the absence of a feeder layer) was the initial density of the fibroblasts. We determined the optimal density to be 1×10^4^–5×10^4^ (depending on the growth rate of each single line) fibroblasts per single well of a six-well plate. Fibroblasts were seeded on gelatin on day 0 and received daily transfections of a cocktail of five mRNAs encoding for the transcription factors *OCT3/4, SOX2, KLF4, c-MYC* and *LIN28* (OSKML) at the molar ratio of 3∶1∶1∶1∶1 for 8 to 12 consecutive days as described in Figure 1. Importantly we used the native versions of the five transcription factors as opposed to Warren et al., 2012 where six reprogramming factors (addition of *NANOG*) and a modified version of *OCT3/4* [16] (addition of an N-terminal transactivation MyoD domain) were used. Unlike previous protocols, the dosage of RNA was kept constant over the reprogramming experiment. Titration of initially plated cells combined with a strict quality check of *in vitro* transcribed mRNAs proved to be essential aspects of the protocol [9]. These changes resulted in a less complex, more streamlined and efficient protocol, minimizing technical variability. Using this optimized protocol we established RiPSCs clones as early as day seven in the absence of any feeder layer and by using xeno-free culture media (Pluriton, Stemgent, Cambride, MA). Cultures were maintained under hypoxic conditions (5%), as this has been shown to promote reprogramming [17]. Three to five days after the initial transfection, fibroblasts began to acquire a compact, epithelioid morphology (Figure 1). Live staining of cell surface markers TRA-1-60 and TRA-1-81 assisted in identification and tracking of emerging colonies (Figure S1) and we calculated reprogramming efficiencies between 0.2–4% based on TRA-1-60 positive stained colonies. Three to ten fully reprogrammed hESC-like colonies (identified by morphology and expression of TRA-1-60 and/or TRA-1-81) were mechanically picked between day 10 and 16, clonally expanded in a 1∶1 blend of mTeSR1 and Nutristem, and characterized (Table S1). Here, we present data from one representative mRNA induced pluripotent stem cell clone obtained from each of the four original fibroblast lines: RiPSC.BJ, RiPSC.HUF1, RiPSC.HUF58 and RiPSC.GM13325.

### Characterization of RiPSCs

**Figure 2:**
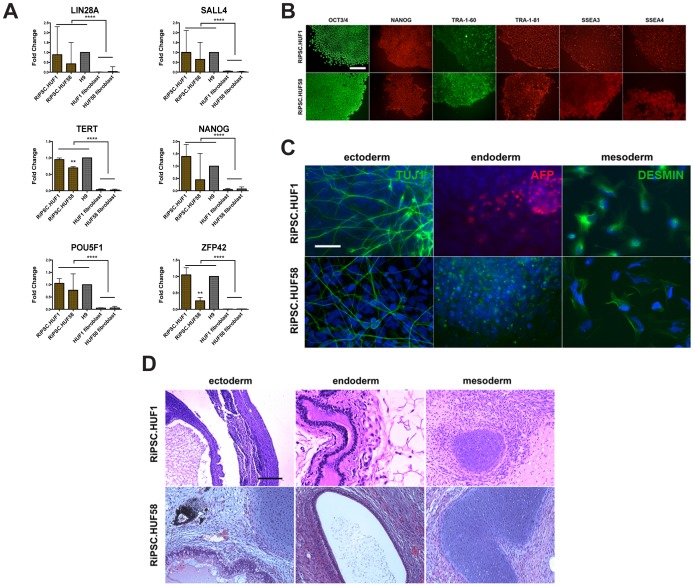
Characterization of RiPSC lines in research conditions. (**A**) Gene expression analysis in RiPSC.HUF1 and RiPSC.HUF58. Expression of 6 markers (normalized to geometric mean of housekeeping genes GAPDH, ACTB, RPLP0, HSP90AB1, HPRT1) was analyzed and compared to the expression levels of human embryonic stem cells (H9) and of the parental fibroblast line. Y-axis shows fold changes compared to H9. Significant differences between fibroblast and pluripotent lines are presented. **** with p<0.0001. Significant differences between RiPSC.HUF58 and RiPSC.HUF1/H9 lines are shown. ** with p<0.01. Data are represented as mean ± SEM. Gene expression data values were determined using Fluidigm technology. (**B**) Immunocytochemistry of two transcription factors (OCT3/4 and NANOG) and four surface markers (TRA-1-60, TRA-1-81, SSEA3, and SSEA4) in expanded RiPSC clones (RiPSC.HUF1 and RiPSC.HUF58). Scale bar = 200 μm. (**C**) Immunocytochemistry showing expression of the lineage markers AFP (alpha-fetoprotein, endodermal), DESMIN (mesodermal) and TUJ1 (Beta III tubulin, neuroectodermal) in *in vitro* differentiated RiPSC clones. Scale bar = 250 μm. (**D**) Hematoxylin and eosin staining of RiPSC.HUF1 and RiPSC.HUF58 derived teratomas showing ectoderm (neural rosettes, epidermis), mesoderm (cartilage) and endoderm (gut-like endothelium). Scale bar = 200 μm. See also Figure S2 and Table S1.

Pluripotency of RiPSCs was characterized by classic assays. Immunocytochemistry revealed strong expression of the transcription factors OCT3/4 and NANOG as well as the surface markers SSEA3, SSEA4, TRA-1-60 and TRA-1-81 in undifferentiated cells (Figure 2B and S2C). Gene expression analysis demonstrated that established RiPSC clones have a molecular signature similar to hESCs (H9). All markers of pluripotency that were examined in H9, RiPSCs and in the original fibroblast lines (Figure 2A and Figure S2A) demonstrated significantly higher expression in RiPSCs and H9 relative to parental fibroblast lines. Notably, RiPSC.HUF58 revealed significantly less expression of TERT and ZFP42 compared to other pluripotent lines. In addition, analysis of differentiation of RiPSC.BJ, RiPSC.HUF1, RiPSC.HUF58, and RiPSC.GM13325 indicated contribution to all three germ layers *in vitro*, as confirmed via immunocytochemistry (Figure 2C and Figure S2D). Results were confirmed *in vivo* in teratoma assays after subcutaneous injection or kidney capsule transplantation into SCID mice (Figure 2D and Figure S2E). Finally, RiPSCs displayed a normal karyotype (Figure S2B). Taken together these results demonstrate efficient derivation of RiPSCs from adult dermal and newborn foreskin fibroblasts under feeder-free conditions. RiPSCs are fully reprogrammed to an undifferentiated state and characterized by classic assays including contribution to all three germ layers *in vivo*. Importantly all the assays were run on iPSC clones at early passage numbers (passage 15) suggesting that our derivation protocol can give high quality iPSCs clones in a short window of time.

### Conversion of research-grade RiPSCs to putative GMP-grade RiPSCs

**Figure 3:**
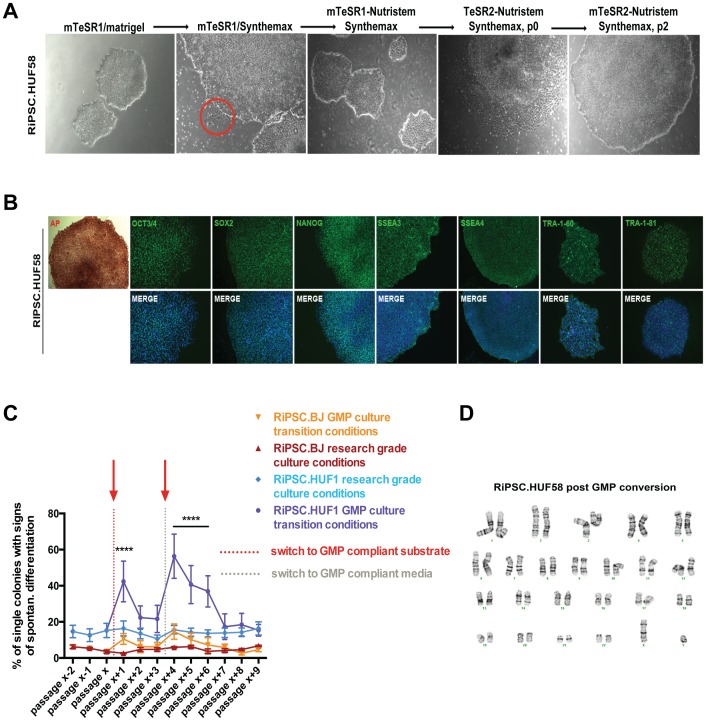
Characterization of RiPSC lines in GMP conditions. (**A**) Morphology tracking of RiPSC colonies during different substrate and media environment changes highlighting the difficulties in adaption in TeSR2/Nutristem conditions. Red circle indicates spontaneous differentiation. Scale bar = 200 μm. (**B**) Immunocytochemistry showing expression of a panel of pluripotency markers in converted RiPSC clones (RiPSC.HUF58). Scale bar = 150 μm. (**C**) Quantification of spontaneous differentiation. Two lines (HUF1, BJ) were derived in research-grade conditions and converted to GMP-grade culture conditions at passage x. Two red arrows indicate switch to GMP compliant conditions. Each data point is an N of 70–150 colonies. (**D**) Normal karyotype (46, XY) of RiPSC.HUF58 after successful GMP conversion. See also Table S2.

In order to make our lines GMP-compliant we next converted them to a xeno-free substrate and a fully defined media environment while maintaining a pluripotent phenotype (Figure 3A and Figure S1). Two strategies to fully convert our lines towards GMP-compatible conditions were applied: (1) Cells were first gradually converted to a 1∶1 blend of TeSR2/Nutristem (both xeno-free) and then passaged onto a new substrate (Synthemax). (2) Cells were first passaged onto Synthemax before the media was gradually switched from mTeSR1/Nutristem to a 1∶1 blend of TeSR2/Nutristem. The latter proved to be more feasible and was performed over a period of 14–30 days. Noteworthy, the switch from mTeSR1/Nutristem to TeSR2/Nutristem blend resulted in a drastic change in cell morphology indicating widespread spontaneous differentiation. In order to restrict spontaneous differentiation during the conversion process to a minimum we manually passaged cells in small clumps by retaining a minimum number of cells per clump (200–300). Survival of RiPSCs during passaging and subculture was improved through the addition of rho-kinase inhibitor (Y-27632) to the growth medium prior to passaging [16]. Colonies that retained morphology typical of undifferentiated cells were selected and manually passaged multiple times until a uniform and stable culture of undifferentiated cells was achieved.

In our efforts to quantify spontaneous differentiation we transitioned two lines (BJ, HUF1) from research-grade (matrigel and mTeSR1) towards GMP-grade (Synthemax and 50/50 blend of Nutristem/TeSR2) and counted spontaneous differentiation events as follows: cells were thawed and manually passaged three times prior GMP-conversion. Manual passaging resulted in relatively equal sized small cell clumps and media was changed for 4 days. Before each manual passage RiPS colonies were counted (70–150 colonies) and the fraction of those colonies with signs of spontaneous differentiation was calculated. Experiments were performed with three biological replicates. The percentage of spontaneously differentiated colonies was calculated prior to each manual passage throughout the conversion process. We applied our second strategy of GMP conversion (passage onto Synthemax first followed by media switch) as this proved to be more feasible.

Results indicate that BJ cells are more stable than HUF1 prior and during GMP conversion with respect to maintaining an undifferentiated morphology (Figure 3C). Particularly, about 40% of HUF1 colonies spontaneously differentiated after passage onto the new substrate (Synthemax, vertical red dashed line), which was significantly higher than cells that were kept in research-grade conditions. BJ cells also showed significantly more colonies with spontaneous differentiation after the switch to Synthemax. Cells adapted to the new conditions as they gradually decreased the number of differentiated colonies. A second spike in spontaneous differentiated colonies was observed upon media change (vertical grey dashed line), which resulted in even more spontaneously differentiated colonies for both lines (HUF1: about 50% differentiated, BJ: about 15% differentiated). BJ cells fully adapted to GMP-compliant conditions after 5 passages as they showed similar percentages of spontaneously differentiated colonies compared to research-grade conditions. HUF1 cells (that initially had a higher potential to spontaneously differentiate in research-grade conditions) needed 7–9 passages to reach levels of spontaneous differentiated colonies in research-grade conditions.

**Figure 4:**
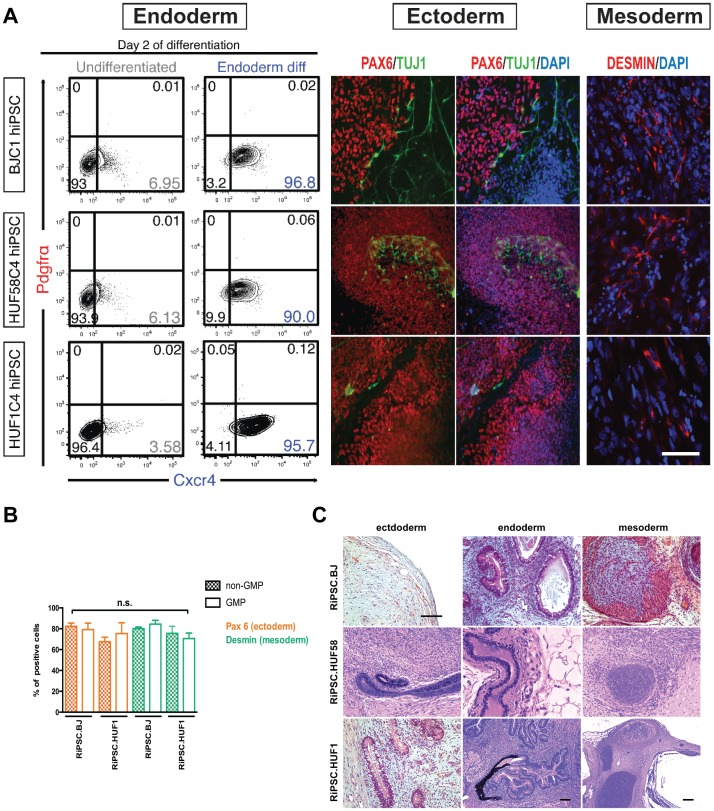
*In vitro* and *in vivo* differentiation of GMP-transitioned RiPSC lines. (**A**) Directed *in vitro* differentiation of three GMP-transitioned RiPSC lines into three germ layers followed by FACS analysis (endoderm) and immunocytochemistry (ectoderm, mesoderm). Scale bar = 150 μm. (**B**) Comparison of differentiation potential into neuroectoderm (PAX6) and mesoderm (DESMIN) between non-GMP (research-grade) and GMP-grade lines (BJ, HUF1). Comparisons are based on counting analysis of cells that were positive for the specific differentiation markers. N = 200–300. (**C**) Hematoxylin and eosin staining of teratomas derived from GMP-transitioned lines showing ectoderm (neural rosettes, epidermis), mesoderm (cartilage) and endoderm (gut-like endothelium). Scale bar = 200 μm.

Following this procedure we successfully converted four RiPSC lines (RiPSC.BJ, RiPSC.HUF1, RiPSC.HUF58, and RiPSC.GM13325) to the new media and new substrate within the GMP facility. These cells passed stringent tests for sterility including tests for the absence of Gram positive and negative bacteria, fungi, mycoplasma and endotoxins (Table S2). Expression of key pluripotency markers remained positive (Figure 3B), emphasizing their undifferentiated character after full conversion. Karyotype analysis (Figure 3D) was performed to exclude any chromosomal abnormalities due to the colony selection. Short tandem repeat analysis confirmed the clonal character of our lines and no match of the DNA fingerprint pattern of the cell lines with any other cell published in the ATCC, NIH or DSMZ website (Table S2). To exclude phenotypical alterations of GMP converted lines with respect to their differentiation potential we re-evaluated their ability to differentiate both *in vitro* and *in vivo*. All lines were efficiently differentiated directly into the three germ layers (Figure 4A and Figure 3SA), formed beating cardiomyocytes (Video S1–S3) and formed all three germ layers upon teratoma formation *in vivo* (Figure 4C). Interestingly, GMP converted RiPSC.BJ cells resulted in substantially less beating cardiomyocytes compared to RiPSC.HUF1 and RiPSC.HUF58. Moreover, we tested whether the highly defined GMP conditions could have an impact on differentiation efficiencies into two lineages – ectoderm and mesoderm by comparing two lines (RiPSC.HUF1, RiPSC.BJ) before and after GMP conversion. Our results indicate that GMP-transitioned lines did not reveal noticeable differences between non-GMP and GMP-compliant cultured RiPSC lines and their potential to form DESMIN+ (mesoderm) and PAX6+ (neuroectoderm) cells (Figure 4B).

Taken together, we demonstrate that our protocol of derivation of RiPSCs can be used to derive pluripotent lines that could be successfully transferred into a GMP facility and that could also pass the GMP compliance tests for their identity, purity, safety and stability (Table S1–S3).

### Derivation of RiPSCs under fully defined culture conditions

**Figure 5:**
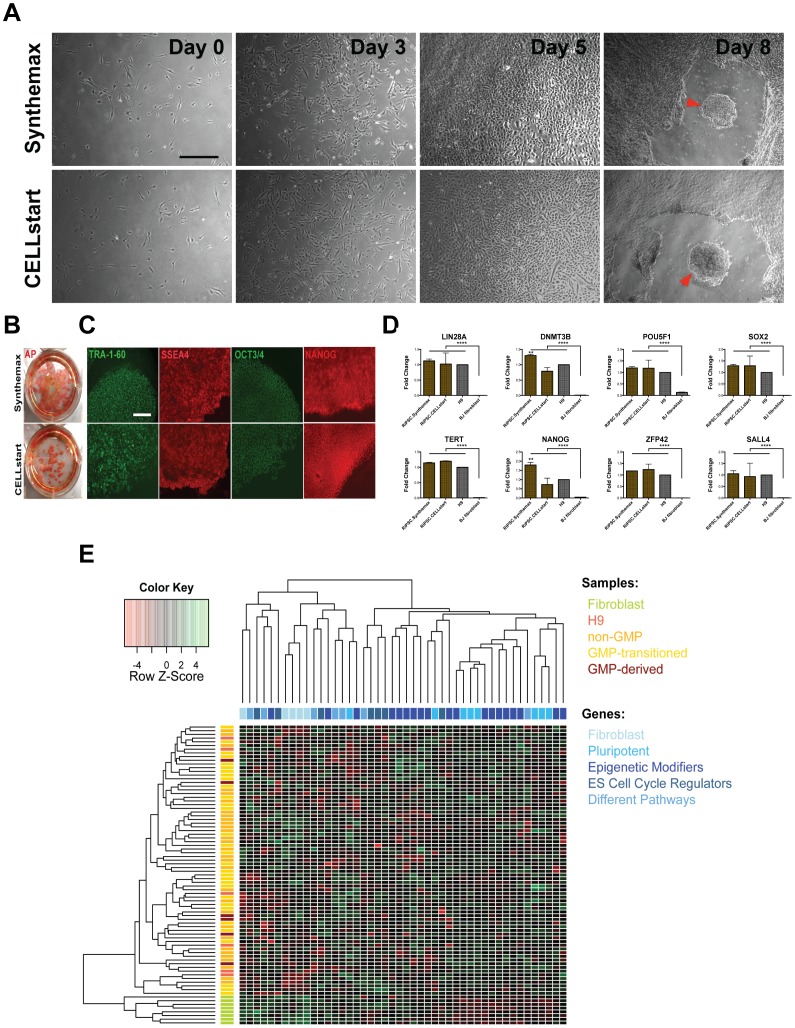
Derivation and characterization of RiPSC.BJ under fully defined GMP grade culture conditions. (**A**) Derivation of RiPSC.BJ on xeno-free, GMP compatible culture conditions on two different substrates: CELLstart and Synthemax. Arrows indicate colony formation after layer of fibroblast has been peeled off by day 8. Scale bar = 180 μm. (**B**) Derivation of RiPSC.BJ on GMP compatible Synthemax and CELLstart matrix followed by alkaline phosphatase staining of parental plates. (**C**) Immunocytochemistry showing expression of a panel for pluripotency markers in RiPSC.BJ cells derived with GMP compatible reagents and matrices. Scale bar = 150 μm. (**D**) Gene expression analysis in RiPSC.BJ dervived on Synthemax or CELLstart. Expression of 8 markers (normalized to geometric mean of housekeeping genes GAPDH, ACTB, RPLP0, HSP90AB1, HPRT1) was analyzed and compared to the expression level of human embryonic stem cells (H9) and of the parental fibroblast line. Y-axis shows fold changes compared to H9. Significant differences between fibroblasts and pluripotent lines are presented. **** with p<0.0001. Significant differences between RiPSC.BJ derived on Synthemax and RiPSC.BJ derived on CELLstart/H9 lines are shown. ** with p<0.01. Data are represented as mean ± SEM. Gene expression data values were determined using Fluidigm technology. (**E**) Unsupervised hierarchical clustering in a heat map plot with 46 different genes. Fibroblast cells revealed a significantly different gene expression profile compared to all pluripotent cells. Clustering failed to discriminate between H9 cells, non-GMP (research-grade), GMP-transitioned and GMP-derived cells highlighting their similar transcriptional profiles. Genes are classified according to their specificity for fibroblasts, pluripotent cells, epigenetic modifiers, ES cell cycle regulators and members of different pathways. See also Figure S3 and Table S3.

Our initial protocol of derivation made use of substrates (porcine gelatin and Matrigel) and media (mTeSR1) which are not free of animal contaminants and which could not be traced for their quality. In an effort to make our protocol even more straightforward and easily reproducible in any GMP environment we attempted the derivation of RiPSCs clones in a GMP compliant manner by using GMP compatible matrices for the initial seeding of BJ fibroblasts and by using only xeno-free Pluriton culture media that had not been conditioned with NuFFs. We tested two different matrices: CELLstart and Synthemax. On both matrices, we derived multiple AP positive iPSCs colonies (∼100–200 on Synthemax and ∼50–100 on CELLstart, Figure 5A, 5B) as early as day seven. Colonies were immediately converted to a blend of TeSR2/Nutristem on the corresponding matrices, expanded and characterized between passage 2–5 (Figure 5C and 5D and Figure S3B). Notably, the direct conversion to TesR2/Nutristem resulted in stable cultures of iPSCs that did not show any major sign of differentiation, resulting in xeno-, integration- and feeder-free iPSC lines. Moreover, transcriptional profiling with 46 different genes revealed that they were indistinguishable from research-grade and GMP-converted RiPSCs as well as hESCs (Figure 5E).

## Discussion

The clinical application of hiPSCs requires that their production must meet GMP-compatible standards but also that the derivation of specified cell derivatives (used for transplantation) from pluripotent stem cells is performed under GMP conditions. Ideally the entire process, from the derivation of hiPSCs lines from patients' somatic cells to the differentiation of such lines into transplantable fully differentiated cells, should be performed in a GMP-compliant environment. Nonetheless previous federal oversight has indicated (and accepted in the case of the Geron Inc. clinical trial, Geron 2009) that the conversion of pluripotent stem cell lines from a research-grade environment to a GMP-grade environment is potentially an accepted practice as long as rigorous tests are run on the converted lines to make sure that no detectable contamination of pathogens of animal origin can be found in the cells. Furthermore given the multitude of technologies for hiPSCs derivation developed in the last few years it is of great importance that a simple, fast, efficient, reproducible protocol of derivation is developed that will ensure the derivation of bona fide hiPSCs clones with no integration of foreign DNA and limited accumulation of mutations due to extensive culture.

In this study we have addressed both of the questions mentioned above. We have for the first time converted human iPSCs, derived using modified synthetic mRNAs under research-grade conditions, into putative GMP-grade conditions. Specifically, we slowly transitioned the cells from a xeno-containing substrate and media to xeno-free conditions that maintained the pluripotent character of the hiPSCs, within a GMP-compliant facility and cultured the cells using qualified defined reagents and a standardized protocol [18]. The converted lines maintained a normal karyotype, showed similar differentiation potentials both *in vitro* and *in vivo* compared to non-GMP lines, and were free of measurable contaminants of non-human origin. We achieved a 100% rate of success in the conversion. We anticipate that the availability of these GMP-compliant and fully characterized RiPSC lines will broadly benefit the scientific community because they represent a suitable platform for drug screening and toxicology tests. Furthermore the cells could be potentially used to produce cell derivatives within the GMP environment and following the strict release criteria that the FDA requires for the clinical therapies and therefore represent a useful resource for the scientific community. However, it must be noted that the previous FDA-approved conversion of research-grade human pluripotent stem cells to clinical-grade cells involved embryonic stem cells, not iPSCs, and it is unclear if the same FDA conversion criteria will apply for iPSCs. Also the FDA approval applied to one specific iPSC-derivative (oligodendrocyte precursor cells), and highlights that a separate FDA approval will be required for each therapeutic product derived from the converted iPSCs. Two recent studies have underlined both interest and necessity to convert hiPSCs from research-grade into a putative clinical-grade state; however hiPSCs converted to GMP conditions by one study were initially derived with a genome-integrating method based on a lentiviral vector [12] or have not been converted at all with low reprogramming efficiencies for primary fibroblasts [19]. The clinical applicability of these GMP-converted RiPSCs and their therapeutic derivatives has yet to be determined. Lastly we should also emphasize that further studies will have to be performed to understand how the different methods of reprogramming affect the quality for the hiPSCs (e.g., in the case of mRNA-based reprogramming, the daily transfections and the induced immune response of the cells)

In an effort to develop a more standardized protocol of derivation of integration free iPSCs that could potentially be adopted by any GMP facility for the derivation of hiPSCs, we have also successfully derived RiPSCs clones that are fully pluripotent by the most stringent pluripotency assays to date for human pluripotent cells [20] by making use only of chemically defined matrices and animal-free reagents that are already in use in GMP facilities. Notably, we observed more homogenous gene expression levels of pluripotency markers between biological replicates compared to lines that were derived under research-grade conditions (compare standard deviations). Moreover, a subset of pluripotency markers (DNMT3B, NANOG) was significantly higher in cells that were derived on Synthemax, suggesting that derivation under GMP-grade conditions may affect the quality of the reprogramming.

This newly established protocol greatly surpassed our original protocol and previously published protocols of derivation of RiPSCs being shorter (and therefore less expensive and with a reduced handling of the cells), reproducible (shown to successfully reprogram a cohort of fibroblasts of different ages and genomic make-ups), scalable, and fully defined. Therefore our fully defined protocol is easily adoptable by any GMP facility for derivation of RiPSCs and represents a valuable resource for the establishment of standard operating procedures that could be adopted by GMP facilities.

In conclusion we emphasize that defined practices need to be put in place for manufacturing and using hiPSCs in regenerative medicine. Our data demonstrate in proof of principle fashion the realization of hiPSC under putative GMP-compliant conditions and represents a basis for the future use of hiPSCs in clinical trials.

## Supporting Information

Figure S1 **Related to** **Figure 1:** **TRA-1-60 and TRA-1-81 live staining.** TRA-1-60 and TRA-1-81 live immunostaining during reprogramming used for colony identification. Arrows indicate colony. Scale bar = 150 μm.
(TIF)

Figure S2 **Related to** **Figure 2:** **Characterization of RiPSC lines in research conditions. (A)** Gene expression analysis in RiPSC.GM13325. Expression of 6 markers was analyzed and compared to the expression level in the parental fibroblast line. Data are represented as mean ± SEM. **(B)** Normal karyotype (46, XX) of RiPSC.GM13325. **(C)** Immunocytochemistry of one transcription factor (OCT3/4) and four surface markers (SSEA3, SSEA4, TRA-1-60, and TRA-1-81) in expanded RiPSC clone RiPSC.GM13325. Scale bar = 200 μm. **(D)** Immunocytochemistry showing expression of the lineage markers AFP (alpha-fetoprotein, endodermal), DESMIN (mesodermal) and TUJ1 (Beta III tubulin, neuroectodermal) in *in vitro* differentiated RiPSC clones. Scale bar = 300 μm. **(E)** Hematoxylin and eosin staining of RiPSC.BJ and RiPSC.GM13325 derived teratomas showing ectoderm (neural rosettes), mesoderm (cartilage), and endoderm (gut-like endothelium). Scale bar = 200 μm.
(TIF)

Figure S3 **Related to** **Figure 4** **and** **Figure 5:** ***In vitro*** **differentiation of GMP-transitioned RiPSC lines and** ***in vitro/in vivo*** **differentiation of RiPSC.BJ after derivation in fully defined conditions. (A)** Immunocytochemistry showing expression of the neuroectoderm specific markers (NeuN, TUJ1, NESTIN) and mesoderm specific marker (TROPONIN-T) in GMP-transitioned and *in vitro* differentiated RiPSC clones. Scale bar = 250 μm. **(B)** Top panel: Immunocytochemistry showing expression of the lineage markers AFP (alpha-fetoprotein, endodermal), DESMIN (mesodermal) and TUJ1 (Beta III tubulin, neuroectodermal) in *in vitro* differentiated RiPSC clones. Scale bar = 250 μm. Bottom panel: Hematoxylin and eosin staining of RiPSC.BJ derived teratomas showing ectoderm (neural rosettes), mesoderm (cartilage), and endoderm (gut-like endothelium). Scale bar = 200 μm.
(TIF)

Table S1 **Related to** **Figure 1:** **Fibroblast cell lines used to reprogram into mRNA induced pluripotent stem cells.** Information regarding successful GMP transfer and clone number are indicated.
(DOC)

Table S2 **Related to** **Figure 2:** **GMP-compliant reagents for RiPSC line derivation, culture and cryopreservation.** List of all GMP-compliant reagents that were used in this study.
(DOC)

Table S3 **Related to** **Figure 2:** **RiPSC line sterility and pathogen testing after GMP conversion.** RiPSC lines of this study were tested for various pathogens according to GMP guidelines.
(DOC)

Video S1 **Beating cardiomyocytes after** ***in vitro*** **differentiation of RiPSC.BJ line**. Cells were differentiated while attached to the culture plate.
(MPG)

Video S2 **Beating cardiomyocytes after** ***in vitro*** **differentiation of RiPSC.HUF1 line.** Cells were differentiated while forming EBs.
(M4V)

Video S3 **Beating cardiomyocytes after** ***in vitro*** **differentiation of RiPSC.HUF58 line.** Cells were differentiated while forming EBs.
(M4V)
