# Supplementary figures and images for: Rapid and Efficient Conversion of Integration-Free Human Induced Pluripotent Stem Cells to GMP-Grade Culture Conditions
Source: PLoS One. 2014 Apr 9;9(4):e94231. doi: 10.1371/journal.pone.0094231 (PMC3981795; doi:10.1371/journal.pone.0094231)

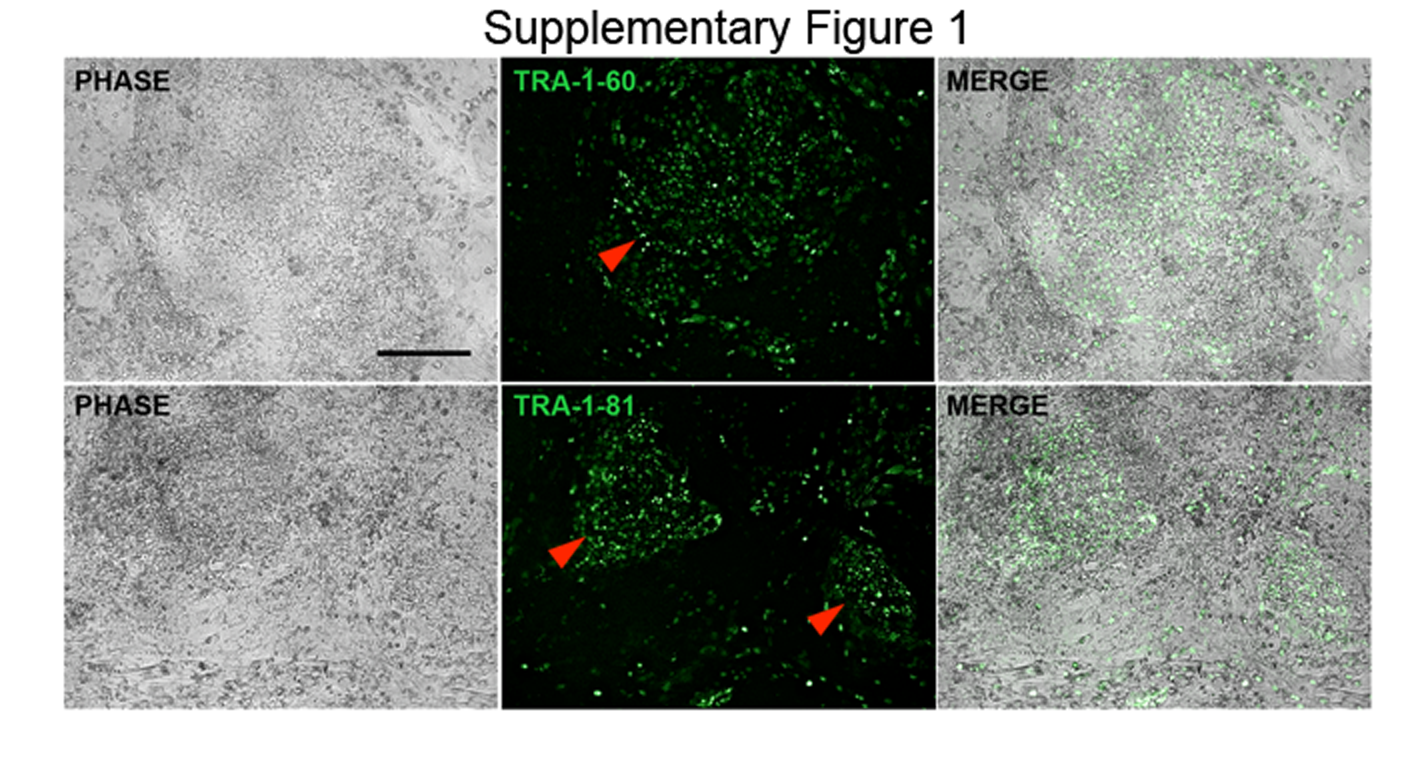

Supplement: Figure S1 — Related to Figure 1: TRA-1-60 and TRA-1-81 live staining. TRA-1-60 and TRA-1-81 live immunostaining during reprogramming used for colony identification. Arrows indicate colony. Scale bar = 150 μm. (TIF) [file pone.0094231.s001.tif]

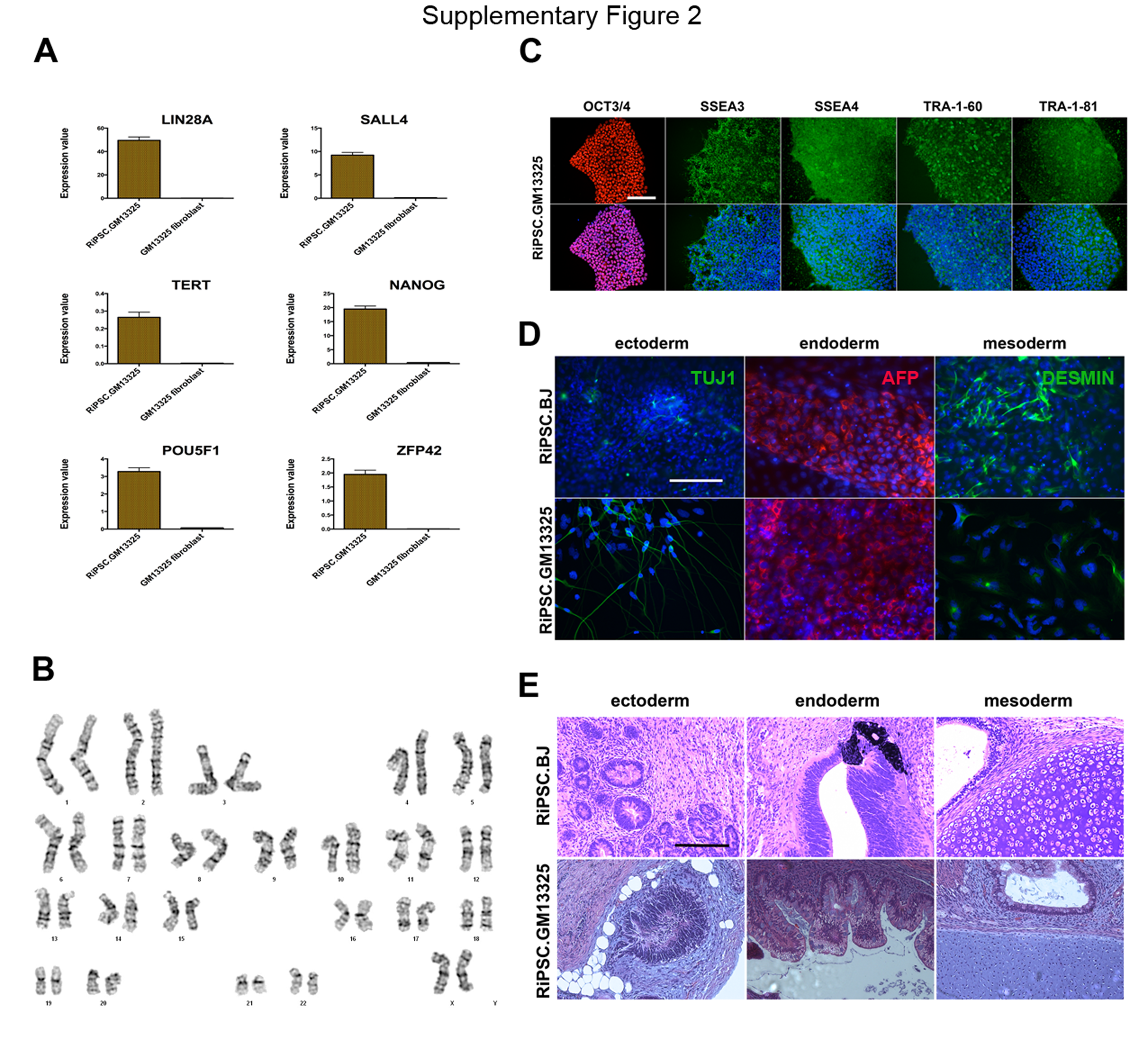

Supplement: Figure S2 — Related to Figure 2: Characterization of RiPSC lines in research conditions. (A) Gene expression analysis in RiPSC.GM13325. Expression of 6 markers was analyzed and compared to the expression level in the parental fibroblast line. Data are represented as mean ± SEM. (B) Normal karyotype (46, XX) of RiPSC.GM13325. (C) Immunocytochemistry of one transcription factor (OCT3/4) and four surface markers (SSEA3, SSEA4, TRA-1-60, and TRA-1-81) in expanded RiPSC clone RiPSC.GM13325. Scale bar = 200 μm. (D) Immunocytochemistry showing expression of the lineage markers AFP (alpha-fetoprotein, endodermal), DESMIN (mesodermal) and TUJ1 (Beta III tubulin, neuroectodermal) in in vitro differentiated RiPSC clones. Scale bar = 300 μm. (E) Hematoxylin and eosin staining of RiPSC.BJ and RiPSC.GM13325 derived teratomas showing ectoderm (neural rosettes), mesoderm (cartilage), and endoderm (gut-like endothelium). Scale bar = 200 μm. (TIF) [file pone.0094231.s002.tif]

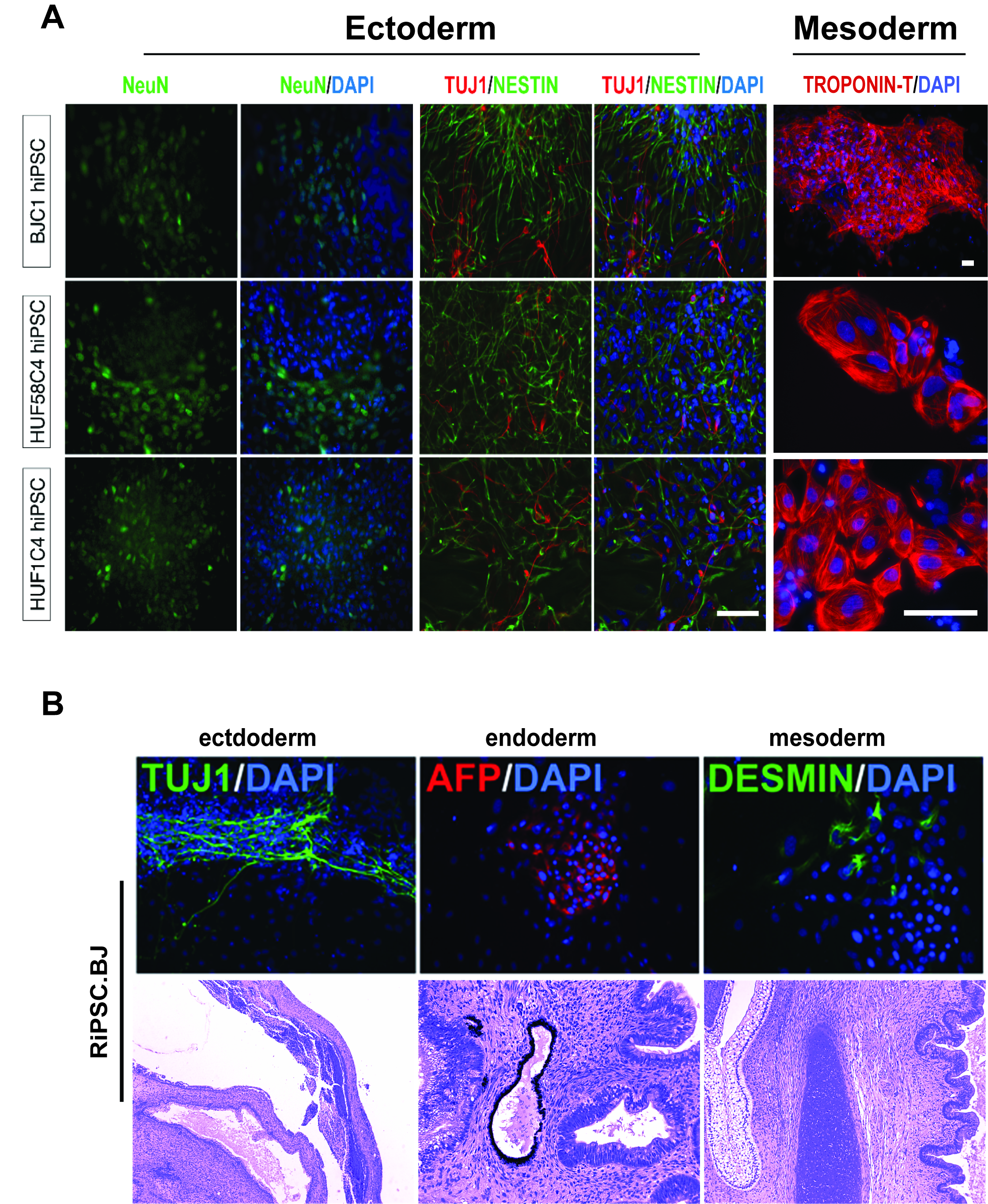

Supplement: Figure S3 — Related to Figure 4 and Figure 5: In vitro differentiation of GMP-transitioned RiPSC lines and in vitro/in vivo differentiation of RiPSC.BJ after derivation in fully defined conditions. (A) Immunocytochemistry showing expression of the neuroectoderm specific markers (NeuN, TUJ1, NESTIN) and mesoderm specific marker (TROPONIN-T) in GMP-transitioned and in vitro differentiated RiPSC clones. Scale bar = 250 μm. (B) Top panel: Immunocytochemistry showing expression of the lineage markers AFP (alpha-fetoprotein, endodermal), DESMIN (mesodermal) and TUJ1 (Beta III tubulin, neuroectodermal) in in vitro differentiated RiPSC clones. Scale bar = 250 μm. Bottom panel: Hematoxylin and eosin staining of RiPSC.BJ derived teratomas showing ectoderm (neural rosettes), mesoderm (cartilage), and endoderm (gut-like endothelium). Scale bar = 200 μm. (TIF) [file pone.0094231.s003.tif]
