# Supplementary material for: Rapid and Efficient Conversion of Integration-Free Human Induced Pluripotent Stem Cells to GMP-Grade Culture Conditions
Source: PLoS One. 2014 Apr 9;9(4):e94231. doi: 10.1371/journal.pone.0094231 (PMC3981795; doi:10.1371/journal.pone.0094231)
Supplement: Table S1 — Related to Figure 1: Fibroblast cell lines used to reprogram into mRNA induced pluripotent stem cells. Information regarding successful GMP transfer and clone number are indicated. (DOC) [file pone.0094231.s004.doc]

| **Cell line** | **Karyotype** | **Age** | **Origin** | **Phenotype** | **Passage # of clones** | **Derived clones** | **Immuno-Phenotype** | **Gene expression** | ***In vitro***  **differentiation** | ***In vivo***  **differentiation** | **Successful GMP transfer** |
| --- | --- | --- | --- | --- | --- | --- | --- | --- | --- | --- | --- |
| BJ | XY | Newborn | Foreskin | WT | 1-35 | 10 | OCT3/4, TRA-1-60, TRA-1-81, SSEA3, SSEA4 | OCT3/4, LIN28A, NANOG, TERT, ZFP42, SALL4 | AFP, TUJ1, DESMIN, PAX6, NESTIN, NeuN, TROPONIN-T, Cardiomyocytes | ✔ | YES |
| HUF1 | XY | 28 years | Dermis | WT | 6-30 | 3 | OCT3/4, NANOG, TRA-1-60, TRA-1-81, SSEA3, SSEA4 | OCT3/4, LIN28A, NANOG, TERT, ZFP42, SALL4 | AFP, TUJ1, DESMIN, PAX6, NESTIN, NeuN, TROPONIN-T, Cardiomyocytes | ✔ | YES |
| HUF58 | XY | 60 years | Dermis | Chromosome 2 pericentric inversion | 4-30 | 4 | OCT3/4, NANOG, TRA-1-60, TRA-1-81, SSEA3, SSEA4 | OCT3/4, LIN28A, NANOG, TERT, ZFP42, SALL4 | AFP, TUJ1, DESMIN, PAX6, NESTIN, NeuN, TROPONIN-T, Cardiomyocytes | ✔ | YES |
| GM13325 | XX | 9 days | Dermis | DiGeorge Syndrome del(22)(q11) | 5-22 | 4 | OCT3/4, TRA-1-60, TRA-1-81, SSEA3, SSEA4 | OCT3/4, LIN28A, NANOG, TERT, ZFP42, SALL4 | AFP, TUJ1, DESMIN | ✔ | YES |

**Supplementary Table S1; Related to Figure 1. Fibroblast cell lines used to reprogram into mRNA induced pluripotent stem cells.** Information regarding successful GMP transfer and clone number are indicated. N.D.= not determined.
