# Supplementary material for: Rapid and Efficient Conversion of Integration-Free Human Induced Pluripotent Stem Cells to GMP-Grade Culture Conditions
Source: PLoS One. 2014 Apr 9;9(4):e94231. doi: 10.1371/journal.pone.0094231 (PMC3981795; doi:10.1371/journal.pone.0094231)
Supplement: Table S2 — Related to Figure 2: GMP-compliant reagents for RiPSC line derivation, culture and cryopreservation. List of all GMP-compliant reagents that were used in this study. (DOC) [file pone.0094231.s005.doc]

| **Reagent** | **Supplier** | **Catalog Number** | **Comments** |
| --- | --- | --- | --- |
| Synthemax | Corning, USA | 3535XX1 | Manufactured under cGMP |
| CELLstart | Invitrogen, USA | A10142-01 | Manufactured under cGMP |
| Pluriton medium | Stemgent, USA | 00-0070 | Xeno-free |
| mTeSR1 | StemCell Technologies, USA | 05850 | Manufactured under cGMP |
| TeSR2 | StemCell Technologies, USA | 05860 | Manufactured under cGMP |
| Nutristem | Stemgent, USA | 01-0005 | Xeno-free |
| mRNA | Stemgent/in house | 00-0067 | Manufactured under defined SOPs |
| NuFF | Globalstem | GSC-3006C | Manufactured under defined SOPs, quality and sterility testing |
| B18R | eBioscience | 34-8185 | Manufactured under cGMP, ASR, or ISO CE-IVD regulatory requirements |

**Supplementary Table S2; Related to Figure 2. GMP-compliant reagents for RiPSC line derivation, culture and cryopreservation.**
