# Supplementary material for: Rapid and Efficient Conversion of Integration-Free Human Induced Pluripotent Stem Cells to GMP-Grade Culture Conditions
Source: PLoS One. 2014 Apr 9;9(4):e94231. doi: 10.1371/journal.pone.0094231 (PMC3981795; doi:10.1371/journal.pone.0094231)
Supplement: Table S3 — Related to Figure 2: RiPSC line sterility and pathogen testing after GMP conversion. RiPSC lines of this study were tested for various pathogens according to GMP guidelines. (DOC) [file pone.0094231.s006.doc]

| **Cell line** | **Sterility** | **Mycoplasma** | **DNA fingerprint** | **Gram+ bacteria** | **Gram- bacteria** | **Fungi** |
| --- | --- | --- | --- | --- | --- | --- |
| RiPSC.HUF58 | neg. | neg. | passed | neg. | neg. | neg. |
| RiPSC.GM13325 | neg. | neg. | passed | neg. | neg. | neg. |
| RiPSC.BJ | neg. | neg. | passed | neg. | neg. | neg. |
| RiPSC.HUF1 | neg. | neg. | passed | neg. | neg. | neg. |

**Supplementary Table S3; Related to Figure 2. RiPSC line sterility and pathogen testing after GMP conversion**
